# Farm Colony Scheme Started

**Published:** 1920-02-07

**Authors:** 


### FARM COLONY SCHEME STARTED.

Three classes of disabled soldiers and sailors call for consideration, because they cannot return to the old ways of life—the blind, because they have to make a new way in a dark world; the limbless, because they have to adjust themselves to fresh conditions of life and work; and the tuberculous, because they have to go through a long cure, and need frequently to find new employment.

The National Association for the Prevention of Consumption, of which Lord Balfour of Burleigh is Chairman, have issued an appeal for support of their scheme to raise £50,000 for a farm colony.

Disappointment has often been expressed at the failure of hospital and sanatorium, when cases that left much improved have fallen back on their return to the old conditions of home and work. The one remedy is to complete the cure and secure its permanence by adaptation and education.

Hospital and sanatorium treatment may arrest the disease, but experience shows that robust health can only be re-established by graduated physical work under proper conditions for a sufficient length of time—this is the first care of the colony.

Many patients can never return to their old employment with safety. To teach them some suitable new employment is the next duty of the colony.

The Association declares that the farm colony is a proved success. For example, the Royal Victoria Farm Colony, near Edinburgh, founded by Sir Robert Philip in 1910, in four years passed out eighty-eight fit men to employments, for the most part, new to them. At least fourteen of them have been with the Colours throughout the war, and have stood the trying campaign well.

The farm colony for sailors and soldiers aims at completing the cure of suitable patients from hospitals and sanatoria, helping them to work out their salvation under proper conditions, training those who require it in some open-air, healthy occupation suited to their condition, especially fitting them for a life on the land, that they may become once more self-supporting citizens.

The Association has determined that the time is ripe for a great effort to establish a national farm colony on these lines for the reception of sailors and soldiers broken down through tuberculosis.

For this purpose the Council of the Association has acquired 115 acres at Frimley, in Surrey. Operations have already begun, and it is hoped the colony will be in working order next summer. The Council have been assured of the warm sympathy of the Pensions Ministry and the cordial co-operation of the leading medical authorities.

The King and Queen and Queen Alexandra are among those who have subscribed the £26,000 so far received. It should be a duty and a privilege to help.

